# Urogynecological and Sexual Functions after Vecchietti Reconstructive Surgery

**DOI:** 10.1155/2019/2360185

**Published:** 2019-02-25

**Authors:** Aneta Adamiak-Godlewska, Katarzyna Skorupska, Tomasz Rechberger, Katarzyna Romanek-Piva, Paweł Miotła

**Affiliations:** 2nd Department of Gynecology, Medical University in Lublin, ul. Jaczewskiego 8, 20-954 Lublin, Poland

## Abstract

**Hypothesis/Aims of Study:**

Mayer–Rokitansky–Küster–Hauser (MRKH) syndrome is the second most common cause of primary amenorrhea. The ESHRE/ESGE categorizes this disorder within the class 5 uterine malformation of the female genital tract anomalies. It is characterized by congenital absence of the uterus, cervix, and upper part of the vagina in otherwise phenotypically normal 46XX females. These patients have normal ovaries, biphasic ovarian cycle, and female psychosexual identification. Laparoscopic Vecchietti's operation—surgical method in which the vagina increases in size by gradually applying traction to the vaginal vault—is one of the methods used to treat MRKH. The aim of this study was to establish the urogynecological and sexual functions after Vecchietti's operation.

**Study Design, Materials and Methods:**

Fifteen patients with MRKHS who underwent laparoscopic Vecchietti's operation were included. A control group of 15 age-matched, childless, sexually active women were examined during the same period. All patients underwent the basic evaluation of anatomical outcomes. Sexual outcomes were established by the Polish validated Female Sexual Function Index (FSFI) questionnaire. Continence status was assessed by Polish validated Urinary Distress Inventory (UDI-6) and the Incontinence Impact Questionnaire (IIQ-7).

**Results:**

Mean age of MRKH group was 22.06±5.13 yrs. Mean follow-up after surgery was 8.02±3.43 yrs. Mean age of women from control group was 22.4±4.35. Mean FSFI scores show good quality of sexual life in both groups. UDI-6 scores showed that patients after Vecchietti surgery have urogynecological problems significantly more often than healthy women do. Based on the IIQ-7, it is evident that one patient from the MRKH group (6,6%) suffers from stress urinary incontinence and the rest (20%) have rather irritative problems with the functioning of the lower urinary tract.

**Conclusion:**

Quality of sexual life after the Vecchietti's operation in long-term follow-up does not differ from that of healthy women, but these patients suffer more frequent from urogynecological complaints. The trial is registered with NCT03809819.

## 1. Introduction

Mayer–Rokitansky–Küster–Hauser syndrome (MRKHS) is the second most common cause of primary amenorrhea. Indeed, the European Society of Human Reproduction and Embryology (ESHRE) and the European Society for Gynaecological Endoscopy (ESGE) categorize this disorder within the class 5 uterine malformation category of the female genital tract anomalies [1]. It is characterized by the congenital absence of the uterus, cervix, and upper part of the vagina in otherwise phenotypically normal 46XX females [2]. These patients have normal ovaries, biphasic ovarian cycles, and female psychosexual identification. While vaginal aplasia is detectable with the physical examination of babies, it is usually diagnosed during adolescence with primary amenorrhea, and, rarely, in the beginning of the sexual life, as complaints of dyspareunia or unsuccessful intercourse [3]. Laparoscopic Vecchietti's operation—a surgical method in which the vagina is increased in size by steadily applying traction to the vaginal vault—is one of the methods used to treat MRKH [4]. This approach involves gentle stretching of the patient's own vaginal skin. An oval device is placed on the vaginal dimple and drawn up gradually by threads that run through the oval from the perineum into the pelvis and out through the abdomen, where they are attached to a traction device. To create a neovagina, the tension is increased on the traction device to pull the thread and stretch the vagina by approximately 1 to 1.5 cm/d until the vagina reaches approximately 7-8 cm in depth [4]. Postoperative management including repeated dilatation of the vaginal dimple is established at least for 6 months: during initial 3 months the dilator is placed on the vaginal dimple using firm pressure for 10 minutes two times a day, for successive 3 months 2-3 times per week. Treatment with dilators was ceased when it was successful; satisfactory intercourse was achieved. Initial supervision and education of proper dilatation were essential to avoid the urethra and anus dilatation [5]. The created neovagina is then covered by nonkeratinized squamous epithelium. MRKHS compromises sexual life and makes natural reproduction impossible. Moreover, associated upper urinary tract malformations are found in approximately 30% of all cases of MRKHS. Among these are unilateral renal agenesis, ureter malformation, ectopia of one or both kidneys, renal hypoplasia, horseshoe kidney, and hydronephrosis [6]. Available literature is insufficient in the area of urogynecological dysfunction such as urinary incontinence in patients treated for MRKHS.

The aim of this study was to establish the degree of urogynecological and sexual functions after the Vecchietti's operation.

## 2. Materials and Methods

Between 2009 and 2015, thirty-five women underwent Vecchietti's operation performed by the same surgeon in our department. For the purposes of this study, we sent 35 letters inviting them for a check-up. Of these, fifteen patients arrived for examination; hence, fifteen patients with MRKHS who had undergone laparoscopic Vecchietti's operation were included. A control group of 15 age-matched, childless, sexually active women were examined during the same period. Those patients came for routine check-up to our outpatient department. All patients provided written informed consent to participate in the study.

All patients underwent the basic evaluation of anatomical outcomes. Pelvic Organ Prolapse Quantification (POPQ) was assessed for every patient. Additionally, we performed the cough stress test (CST) with comfortably full bladder in supine and standing position—recommended in the evaluation of uncomplicated female patients with the complaint of stress urinary incontinence (SUI) [7]. We determined the patient's bladder volume at the time of CST via an ultrasound bladder scan prior to examination. Test was performed in a range of bladder volumes between 200 and 400 ml.

Urine test strip was also done to determine possible pathological changes in patient's urine.

Sexual outcomes were established by the Polish validated Female Sexual Function Index (FSFI) questionnaire, of 19 questions. These enable an assessment of sexual function over the previous 4 weeks. The subscale scores ranged from 0 to 6, with higher scores indicating better sexual function. Subjects obtaining a total PL-FSFI score of 27.50 or lower were considered to have sexual dysfunction [8].

Continence status was assessed by 2 questionnaires: Polish validated Urinary Distress Inventory (UDI-6) and the Incontinence Impact Questionnaire (IIQ-7). IIQ-7 measures the impact of urinary incontinence on activities, roles, and emotional states, whereas the UDI-6 measures how troubling the symptoms are. UDI-6 consists of 6 items, which are subdivided into 3 domains: IS-irritative symptoms, SS-stress symptoms, and OS-obstructive/discomfort symptoms. IIQ-7 consists of 7 items, which are subdivided into 4 domains: PA, physical activity; TR, travel; SA, social activities; and EH, emotional health. For both UDI-6 and IIQ-7, a higher score equals higher disability (completely compromised by urinary symptoms =100). Over all, these questionnaires result in a 0-400 scale: the greater the score, the more problematic the incontinence [9].

## 3. Statistical Analysis

Statistical analysis was performed using Statistica v. 12.0 software (StatSoft, Poland).* P* values less than 0.05 were considered significant. Variables that were not normally distributed (*P* <0.05 by Shapiro-Wilk test) were analyzed via the Mann-Whitney U test.

## 4. Results

Mean age of MRKH group was 22.06±5.13 yrs. Mean follow-up after surgery was 8.02±3.43 yrs. Mean age of women from the control group was 22.4±4.35.

All patients included in the study did not experience intraoperative complications. During examination, we did not observe evidence of postoperative complications or vaginal scarring. In the study group, vaginal length was 7±1.2 cm and all women were in the POPQ-0 stage. In the control group, all women were also in the POPQ-0 stage.

Urine test strip tests were normal for patients from both groups.

All women were in a stable relationship. 14 women (93%) from the MRKH group declared a satisfactory sexual life and this was confirmed by the FSFI questionnaire results. In the control group, sexual satisfaction was declared by 100% of all patients, but the FSFI results confirmed this in 14 (93%) cases.

FSFI and UDI-6, IIQ-7 results are shown in Table 1.

### 4.1. Interpretation of Results

FSFI scores show good quality of sexual life in both groups. Indeed, women from both groups have scores higher than the mean result for the general Polish population (= 27.5).

UDI-6 scores showed that patients after Vecchietti surgery have urogynecological problems significantly more often than healthy women do. Declared problems started about 7 months after surgery. Based on the IIQ-7, it is evident that one patient from the MRKH group (6,6%) suffers from stress urinary incontinence and the rest (20%) have rather irritative problems with the functioning of the lower urinary tract.

### 4.2. Discussion

Vecchietti's operation is one of the leading treatment options for MRKHS. The resulting neovagina enables women with this syndrome to have sexual intercourse. In our study, we confirmed that this method is well accepted by patients in the aspect of sexual satisfaction; however, urogynecological problems affect this group more often than do the healthy population. Previous studies have evaluated the aspect of megalourethra and urinary incontinence due to urethral coitus [10], but stress urinary incontinence problems after the Vecchietti's operation have been only shown in case reports [11, 12]. We agree with Bianchi et al. that the creation of a neovagina can alter the previous anatomy, modifying the balance in the pelvic floor. This leads to the change in the urethrovesical angle and a lack of suburethral support, allowing hypermobility of the urethra [11]. The aforementioned can be the reasons for urinary incontinence. In our study, however, we also observed irritative problems that cannot be explained by pelvic organ prolapse (POP) or bladder infection. Huebner et al. describe the support structures in pelvis before and after Vecchietti's surgery in magnetic resonance imaging. The support ligaments are present in these women preoperatively and the Vecchietti's procedure simply extends the vagina into these areas. Scar tissue at the apex as a result of the peritoneal tunneling during the Vecchietti procedure might enforce apical support of both cardinal and uterosacral ligaments. Missing scar tissue can explain why prolapse has been described quite often after self-dilatation [13].

The FSFI scale used in our study assesses all aspects of a woman's sexual functioning in 4 domains: desire, excitement, lubrication, and orgasm. In our study, the sexual function self-declared by our study group was good. This is no surprise, as a large majority of published studies show similar results [14–16]. We observed problems in only 1 out of 15 women. She was the oldest participant of the study and was 33 at the time of operation; she was observed for 6 years afterwards and usually declared lack of satisfaction in all aspects of sexual life apart from desire.

## 5. Study Limitations

This study includes only a small group of patients; this is partly the result of a limited population of women with MRKHS. The second reason for an insufficient study group in terms of population is the fact that only 15 out of 35 operated women came for evaluation. Most women are operated on when they are 18 years old; they finish secondary schools and travel to study in different towns and sometimes other countries, so it is difficult to contact them.

## 6. Conclusion

Quality of sexual life after the Vecchietti's operation in long-term follow-up does not differ from that of healthy women, but these patients suffer more frequent from urogynecological complaints.

These findings support the need for further research in a larger study group to assess urogynecological outcomes of the Vecchietti's operation.

## Figures and Tables

**Table 1:** FSFI and UDI-6, IIQ-7 results.

QUESTIONNAIRE GROUP	FSFI Me (min-max)	UDI-6 Me (min-max)	II Q7 Me (min-max)
MRKH (n=15)	29.55 (6.80-32.6)	116.55 (0.00-333.00)	0.00 (0.00-366.3)
CONTROL (n=15)	30.95 (21.9-35.4)	33.3 (0.00-199.8)	0.00 (0.00-133.2)
Test U Mann-Whitney (p)	0.43	0.05	0.34

## Data Availability

The data used to support the findings of this study are available from the corresponding author upon request.
